# Prebiotic Route to Thymine from Formamide—A Combined Experimental–Theoretical Study

**DOI:** 10.3390/molecules26082248

**Published:** 2021-04-13

**Authors:** Lukáš Petera, Klaudia Mrazikova, Lukas Nejdl, Kristyna Zemankova, Marketa Vaculovicova, Adam Pastorek, Svatopluk Civis, Petr Kubelik, Alan Heays, Giuseppe Cassone, Jiri Sponer, Martin Ferus, Judit Sponer

**Affiliations:** 1 J. Heyrovský Institute of Physical Chemistry, Czech Academy of Sciences, Dolejškova 3, CZ 18223 Prague 8, Czech Republic; lukas.petera@jh-inst.cas.cz (L.P.); adam.pastorek@jh-inst.cas.cz (A.P.); svatopluk.civis@jh-inst.cas.cz (S.C.); petr.kubelik@jh-inst.cas.cz (P.K.); alan.heys@jh-inst.cas.cz (A.H.); 2 Institute of Biophysics of the Czech Academy of Sciences, Královopolská 135, CZ 61265 Brno, Czech Republic; mrazikovaklaudia@gmail.com (K.M.); sponer@ncbr.muni.cz (J.S.); 3 Department of Chemistry and Biochemistry, Mendel University in Brno, Zemědělská 1, CZ 61300 Brno, Czech Republic; sponerju@gmail.com (K.Z.); marketa.vaculovicova@mendelu.cz (M.V.); 4 Central European Institute of Technology, Brno University of Technology, Purkynova 123, CZ 61200 Brno, Czech Republic; 5 Institute for Chemical-Physical Processes, Italian National Research Council (IPCF-CNR), Viale Ferdinando Stagno d’Alcontres 37, 98158 Messina, Italy; giuseppe.cassone@ipcf.cnr.it

**Keywords:** formamide, thymine, uracil, prebiotic chemistry, origin of life

## Abstract

Synthesis of RNA nucleobases from formamide is one of the recurring topics of prebiotic chemistry research. Earlier reports suggest that thymine, the substitute for uracil in DNA, may also be synthesized from formamide in the presence of catalysts enabling conversion of formamide to formaldehyde. In the current paper, we show that to a lesser extent conversion of uracil to thymine may occur even in the absence of catalysts. This is enabled by the presence of formic acid in the reaction mixture that forms as the hydrolysis product of formamide. Under the reaction conditions of our study, the disproportionation of formic acid may produce formaldehyde that hydroxymethylates uracil in the first step of the conversion process. The experiments are supplemented by quantum chemical modeling of the reaction pathway, supporting the plausibility of the mechanism suggested by Saladino and coworkers.

## 1. Introduction

The RNA world theory [1,2] represents the perhaps most widely accepted hypothesis for the origin of life. It suggests that there was a period of time on our planet when RNA served both as a carrier of genetic information and as a catalyst that catalyzes its own replication. According to this theory, DNA is a later product of evolution. It was most likely selected by nature to overtake the hereditary function of RNA because of its remarkably higher stability.

In the last few years, we have witnessed an unprecedented step forward in the prebiotic synthesis of nucleic acids building blocks. Simultaneous synthesis of DNA nucleosides and RNA nucleosides in the prebiotic pool has been recently reported [3,4,5,6]. Thus, novel studies rather support the view that the evolutionary history of DNA might be as old as that of RNA.

Synthesis of nucleobases from formamide [7] has been reported by numerous investigations [8,9,10,11,12,13,14,15,16,17,18,19]. In the past, it has been claimed that without catalysts or a radical chemistry, only purine derivatives are formed from formamide upon prolonged thermal treatment at ca. 140–160 °C [8,9,11].

**Figure 1:**
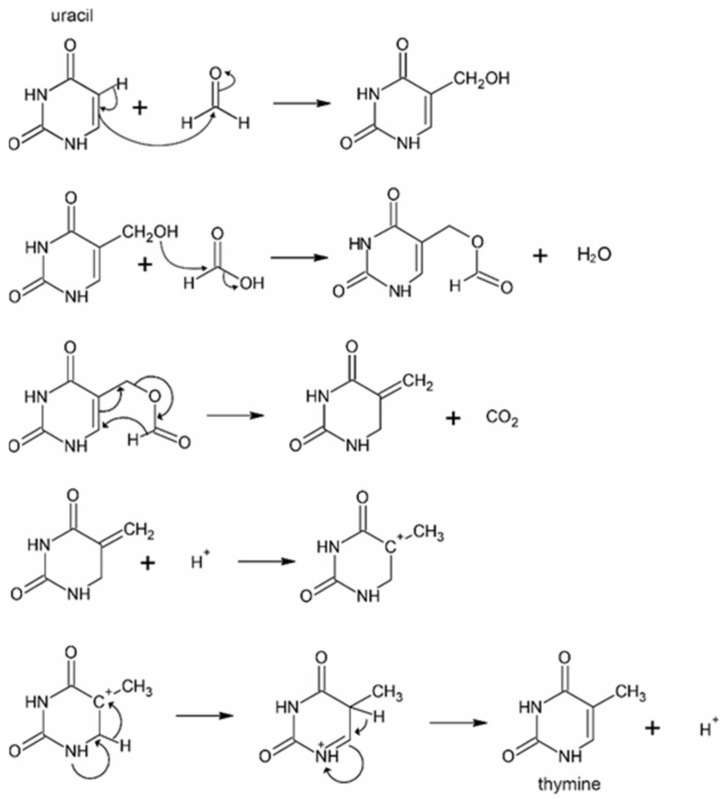
Suggested mechanism for the conversion of uracil to thymine in formamide from [12].

Pioneering investigations by Robertson and Orgel have shown that the C5 position of uracil can easily bind formaldehyde, which enables formation of various C5-substituted uracil derivatives, among them that of thymine [20]. Nevertheless, with the exception of a recent report [21], in formamide medium, formation of thymine [22] has been observed exclusively in the presence of a catalysts [11] or in a radical chemistry [14,15]. For example, Saladino et al. describe formation of thymine in the presence of TiO_2_ [11]. They propose that the role of the TiO_2_ photocatalyst in the reaction is to convert formamide to formaldehyde, which further reacts with uracil, yielding 5-hydroxymethyluracil. The hydroxyl group of this compound then binds formic acid, which subsequently rearranges to thymine upon elimination of CO_2_ (see the reaction scheme shown in Figure 1).

In the current work, using capillary electrophoresis, we reanalyze the products formed upon thermolysis of neat formamide with a particular emphasis on the detection of thymine. We observe that in a lower amount, thymine and other pyrimidine bases may form even in the absence of catalysts in a purely thermal chemistry according to the previously suggested reaction route. We propose that disproportionation of formic acid may play a key role in this chemistry.

## 2. Results and Discussion

### 2.1. Detection of Uracil and Thymine in Heat-Treated Formamide Samples

In the first round of experiments, we prepared four parallel samples that contained pure formamide treated at 160 °C for 24 h. Despite the absence of catalysts, we found that the heat treatment resulted in the formation of detectable amounts of uracil and thymine, which were present in the reaction products in roughly 1 and 0.1 mM concentrations, respectively. The formation of thymine was especially unexpected because the previously suggested pathway for the conversion of uracil to thymine assumes participation of formaldehyde in the conversion. Note that earlier studies [11] associated the presence of formaldehyde in heat-treated formamide solutions with the presence of redox catalysts.

### 2.2. Conversion of Uracil to Thymine in the Presence of Added Formic Acid and Formaldehyde in Formamide Solution

**Figure 2:**
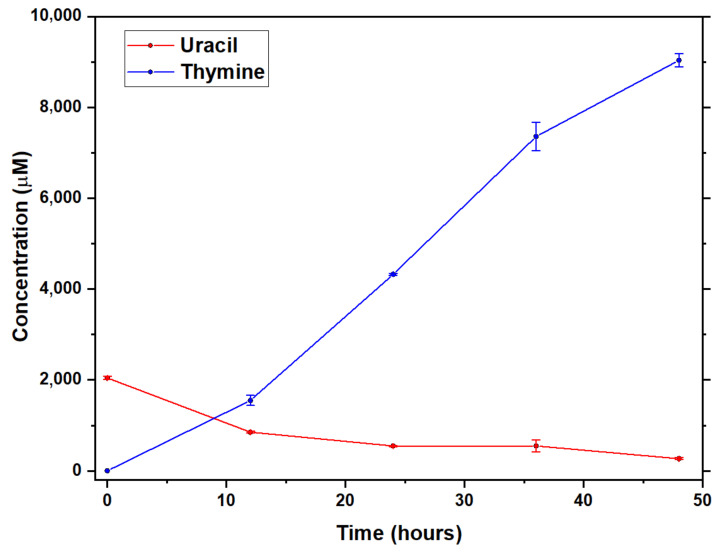
Time dependence of the averaged concentration of thymine and uracil in heat-treated (160 °C) formamide samples containing equimolar amounts of added uracil, formic acid, and formaldehyde (2 mM each) on the beginning of incubation. The experiment was conducted in the absence of added catalysts. Standard deviations (indicated in the graph with error bars) were calculated from at least three independent measurements.

In the next step, we wanted to elucidate whether thymine may form from uracil upon addition of formic acid and formaldehyde as suggested in previous studies [11,12]. For this purpose, we prepared two series of parallel samples that contained equimolar amounts of uracil, formic acid, and formaldehyde (each 2 mM) dissolved in formamide. The samples were incubated in closed glass vials for 0, 12, 24, 36, and 48 h at 160 °C without addition of catalysts. Figure 2 depicts the time dependence of the averaged concentrations of thymine and uracil in the reaction mixture. The figure clearly illustrates that in the first 12 h, thymine production is roughly proportional to the consumption of the initially added uracil. Later, production of thymine unexpectedly accelerates, most likely because a remarkable amount of the formamide solvent is converted to uracil, formic acid, and formaldehyde. Indeed, the uracil concentration is constant until 36 h, while during this period, the concentration of thymine rapidly increases. This suggests that a decisive fraction of the synthesized uracil enters the reaction steps leading to thymine, and that these subsequent steps might have relatively low activation energy under the studied reaction conditions. All these observations suggest that thymine may indeed be formed from uracil possibly according to the mechanism shown in Figure 1.

### 2.3. DFT Modeling of the Conversion of Uracil to Thymine

**Figure 3:**
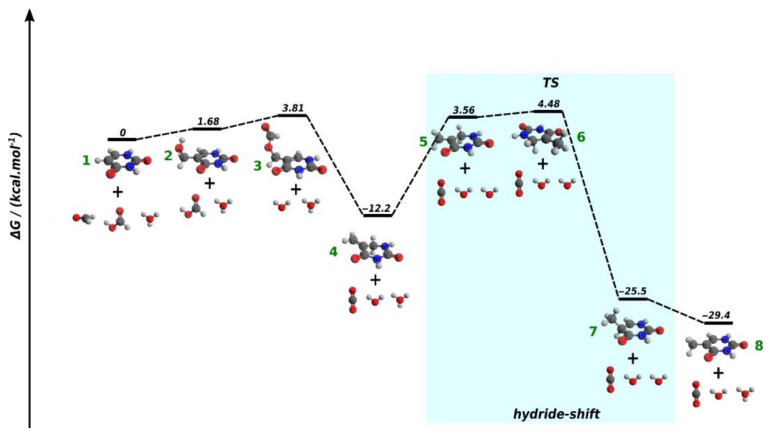
Free energy (ΔG) profile for the conversion of uracil to thymine based on B3LYP/6-31G* calculations. Color coding used in the models: C—dark grey, H—light grey, O—oxygen, N—nitrogen. For Cartesian coordinates of the optimized geometries of compounds **1**-**8**, see the Supplementary Materials.

In order to verify the plausibility of the reaction mechanism for the conversion of uracil to thymine outlined in Figure 1, we carried out quantum chemical calculations. The computed free energy profile of the reaction is depicted in Figure 3. According to our computations, addition of formaldehyde to the C5 position of uracil (**1**) leads to 5-hydroxymethyluracil (**2**) as well as the subsequent esterification step with formic acid proceeds in weakly endothermic reaction steps (endothermicity is within the error margin of the method), which are clearly feasible under constant heating at 160 °C in formamide.

The formate–ester product (**3**) spontaneously loses CO_2_ in a markedly exothermic reaction step leading to **4**. Protonation of the methylene group of **4** in an endothermic reaction step precedes the rate determining step of the whole pathway, which involves a hydride-shift from the C6 carbon of the six-membered ring of **4** to the carbocationic center located at C5 with an activation energy of 16.7 kcal/mol. This activation energy is indeed low; for comparison, the activation energy for the phosphate-catalyzed ring-closure of 2-aminooxazole [23], which is a facile reaction at 60 °C [23], is 23.6 kcal/mol [24] at a similar theoretical level. The product of the hydride shift reaction (**7**) is an N3-protonated thymine, proton loss from the N3-position is accompanied by a free energy change of −4.3 kcal/mol. The overall free energy change for the pathway is −29.4 kcal/mol. Thus, the relatively low activation energy and the clearly exothermic character of the computed mechanism suggest that conversion of uracil to thymine according to the above described chemistry [12] is plausible under the conditions used in our experiments.

### 2.4. Formic Acid and Formaldehyde from Formamide without Added Catalysts

**Figure 4:**
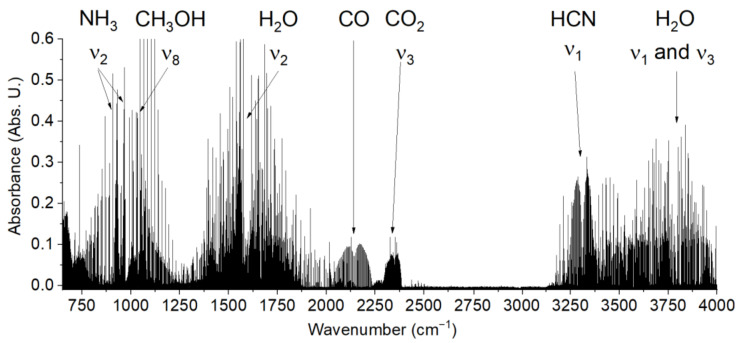
Infrared spectrum of gas phase thermal decomposition products of formamide.

The formamide-based synthesis of relevant prebiotic molecules benefits from the rich repertoire of simple precursors that form upon thermal dissociation of formamide [12,13]. In particular, the synthesis of thymine, beyond uracil, requires formic acid as well as formaldehyde. Formic acid is a well-known product of formamide hydrolysis. Formation of formaldehyde from formamide, however, requires a reductive chemistry. For example, prior to us, it has been observed only in the presence of a TiO_2_ catalyst [11]. Nevertheless, the presence of a large amount of CO_2_ in the gas-phase products of the thermal decomposition of formamide detected by IR spectroscopy (see Figure 4) in our heat-treated samples and in other studies [25] suggests that to a lesser extent, even in the absence of catalysts, some redox processes must take place in the reaction mixture. One possibility for formation of oxidized and reduced C1 compounds from formamide is a disproportionation chemistry, a so far overlooked reaction route. Especially, formic acid is generally considered as a hydride source in synthetic organic chemistry [26,27]. It has been shown that in the presence of catalysts, it may disproportionate producing methanol and CO_2_ [28]. Thus, it is reasonable to expect that to a lesser extent formic acid may serve as a source of reduced C1 compounds (such as formaldehyde), even upon prolonged and harsh heat-treatment of formamide.

## 3. Materials and Methods

### 3.1. Sample Preparation

#### 3.1.1. Formamide Thermolysis

For thermolysis, four parallel samples were prepared in glass flasks. Each sample contained 2 mL of formamide (≥99%, Sigma Aldrich Munich, Germany) and the air atmosphere at the appropriate daily pressure. After filling, the flasks were individually sealed with oxygen–natural gas flame (oxygen; ≥99.995%, Messer Technogas, Bad Soden am Taunus, Germany). All flasks were placed into an oil bath. The oil bath was then under continuous stirring tempered to 160 °C for approximately 2 h. After tempering, the samples were incubated for additional 24 h in the heated oil bath. Subsequently, the samples were removed from the oil bath and allowed to cool to laboratory temperature. Then, all flasks were individually opened by applying a hot quartz rod. Each sample was then transferred into an Eppendorf tube and kept in refrigerator until analysis.

#### 3.1.2. Reaction of Uracil with Formaldehyde and Formic Acid in Formamide Solution

**Figure 5:**
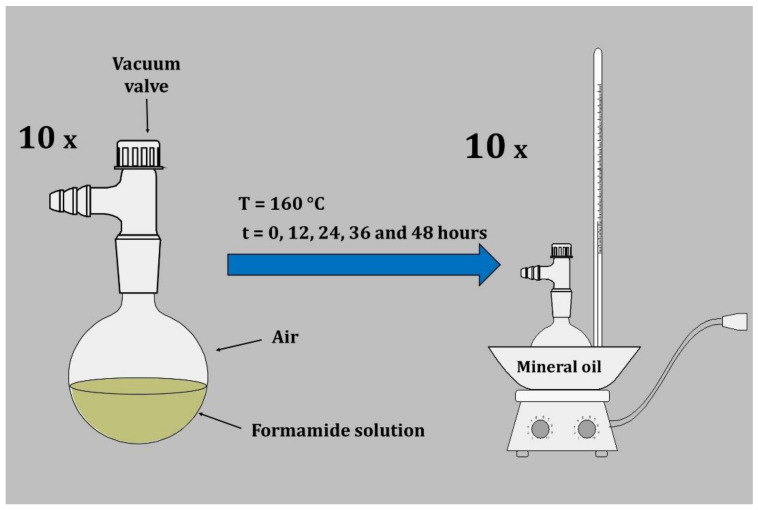
Sketch showing the experimental setup used for thermolysis.

For this purpose, five samples (each in duplicates) were prepared in glass flasks. Each sample contained 2 mL of formamide (≥99%, Sigma Aldrich) and equimolar amounts of dissolved uracil (≥99%, Sigma Aldrich), paraformaldehyde (≥95%, Sigma Aldrich), and formic acid (≥98%, Sigma Aldrich) (each in 2mM concentration). The flasks were filled with air at a total pressure of 500 Torr. All flasks were placed into an oil bath tempered to 160 °C (Figure 5) and were incubated for 0, 12, 24, 36, and 48 h. After removal from the oil bath, the samples were immediately cooled to laboratory temperature and opened. From each sample, the nonvolatile phase was transferred into an Eppendorf tube. The samples were then stored at −28 °C until analysis. A reference pure formamide sample was also prepared and stored in the same conditions.

### 3.2. Micellar Electrokinetic Capillary Chromatography (MEKC)

Quantification of thymine (T) and uracil (U) was performed using a capillary electrophoresis (CE) Instrument 7100 (Agilent Technologies, Waldbronn, Germany) with absorbance detection at a wavelength of 260 nm. A fused silica capillary with an internal diameter of 75 µm, a total length of 64.5 cm, and an effective length of 56 cm was used. The following parameters were used: the sample was introduced hydrodynamically by 40 mbar for 5 s and a separation voltage of 15 kV was applied. A background electrolyte (BGE) was composed of 40 mM borate and 60 mM sodium dodecyl sulfate (SDS) at pH 9.8. Prior to the analysis, the capillary was washed for 100 s using BGE. Before CE analysis, the formamide samples were thawed and adjusted as follows: samples were centrifuged at 12,000 rpm for 10 min, and the supernatant was taken, subsequently diluted 10-fold by distilled water, and then immediately measured using the CE method. In the MEKC electropherograms (see Figure S1 in the Supplementary Materials), the signals corresponding to thymine and uracil can be clearly distinguished based on their typical migration times of 11.5 and 14.3 min, respectively. For more details on the application of the methodology in detection of nucleobases, see [29].

Quantification of uracil and thymine was performed according to the calibration curve and corresponding peak areas. Basic figures of merit of the used method are summarized in Table 1.

### 3.3. Fourier-Transform Infrared (FTIR) Spectroscopy

**Figure 6:**
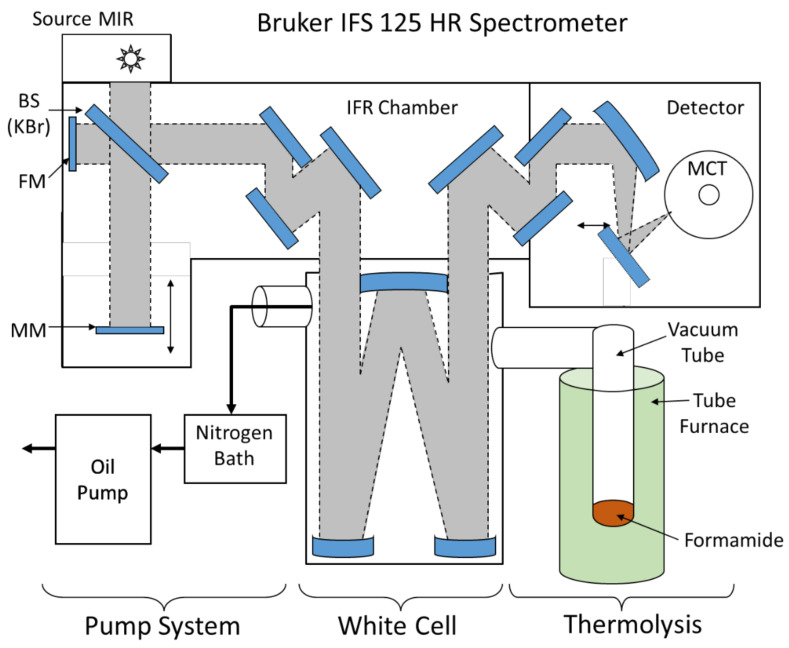
Scheme of the setup used for the inspection of gas phase products formed in the formamide thermal decomposition experiment. BS: beamsplitter, IFR: interferometer, MM: mobile mirror, FM: fixed mirror. Thermolysis was conducted in a vacuum sealed tube inserted in tube furnace.

Gas phase reaction products were examined with Fourier-transform high-resolution spectroscopy using a Bruker IFS 125 HR spectrometer equipped with a KBr beamsplitter and nitrogen-cooled LN-MCT (Hg-Cd-Te) detector. The MCT detector covered the spectral range of 680–4000 cm^−1^. The data were recorded with a spectral resolution of 0.02 cm^−1^ and the number of single scans was set to 300. The sample of formamide was heated under vacuum in electronically controlled tube furnace equipped with Kanthal wire on a ceramic tube up to temperature of 170 °C in vacuum. After 10 min, the gas phase was transferred into a multipass White cell with optical path of 30 m and inspected by FTIR (see Figure 6).

### 3.4. Quantum Chemical (QM) Calculations

Initial geometries for all computed molecules were prepared with Jmol [30]. QM calculations were performed using the Gaussian 09 software [31]. Geometry optimizations and frequency calculations were performed at the B3LYP/6-31G* [32,33,34,35] level of theory with the COSMO implicit solvation model [36] using parameters for formamide solvent. Geometry optimizations employed the Berny algorithm. Stationary point corresponding to the transition state of the hydride shift reaction was located on the potential energy surface using the single coordinate driving (SCD) method. Its transition state character was verified with frequency calculations performed in the harmonic approximation. Free energy (G) data for the computed reaction pathway were calculated from the total electronic energy (E_tot_) and from the thermal and entropic correction terms to the Gibbs free energy (δG) derived from frequency calculations performed in the harmonic approximation at 298 K: *G* = *E*_tot_ + *δG.*

## 4. Conclusions

Earlier papers emphasized the importance of catalysts in the synthesis of pyrimidine nucleobases from formamide [11,12]. In contrast, in the current work, we show that pyrimidine bases, and in particular thymine, do form upon prolonged heat treatment of neat formamide. Our quantum chemical calculations verify the previously suggested mechanism [12] of thymine formation, which involves the conversion of uracil upon hydroxymethylation and subsequent addition of formic acid. The computed activation energy for the pathway by Saladino et al. [12] is 16.7 kcal/mol only, and the rate determining step is associated with the hydride shift reaction initiating the dehydrogenation of the C5-C6 linkage of the six-membered heterocyclic ring (see Figure 1). While previous studies [11,12] find that formation of thymine from uracil in formamide requires the presence of a redox catalyst in the reaction mixture, our results suggest that a noticeable redox chemistry may proceed even in the absence of added catalysts in formamide. The key molecule in this process is formic acid, which is always present in the reaction mixture as the hydrolysis product of formamide. Disproportionation of formic acid may be responsible for the formation of CO_2_ observed with infrared spectroscopy as well as for that of reduced C1 compounds, like formaldehyde. The latter compound plays an indispensable role at the hydroxymethylation of uracil [20], which serves as the initial step on the synthetic way towards thymine.

## Figures and Tables

**Table 1:** Detection limits and linearity for thymine and uracil bases.

Bases	Regression Equation	R	Detection Limit (µM)	Migration Time (min)
Thymine	y = 163.51x − 0.1331	0.999	0.6	11.5
Uracil	y = 216.05x − 0.1199	0.999	0.4	14.3

## Data Availability

Data is contained within the article or Supplementary Material.

## Supplementary Materials

MEKC electropherograms, Figure S1: Separation of nucleobases thymine (T) and uracil (U) by CE (MEKC). BGE: 40 mM sodium borate with 60 mM SDS (pH 9.8). Hydrodynamic injection: 50 mbar for 5 s, separation voltage: 15 kV, absorption signal at λ = 260 nm. Electropherograms (A) pure formamide, (B) 200 μM standards T and U in formamide, (C) formamide sample with 200 μM U before thermolysis (0 h) and (D) after 12 h of thermolysis.
