# Supplementary material for: Prebiotic Route to Thymine from Formamide—A Combined Experimental–Theoretical Study
Source: Molecules. 2021 Apr 13;26(8):2248. doi: 10.3390/molecules26082248 (PMC8069957; doi:10.3390/molecules26082248)
Supplement: Supplementary file 1 [file molecules-26-02248-s001.pdf]

Supplementary material

to the paper

Prebiotic route to thymine from formamide – a combined experimental-theoretical study

by

Lukáš Petera,[1] Klaudia Mráziková,[2] Lukáš Nejdl,[3,4*] Kristýna Zemánková,[3] Markéta Vaculovičová,[3,4] Adam Pastorek,[1] Svatopluk Civiš,[1] Petr Kubelík,[1] Alan Heays,[1] Giuseppe Cassone,[5] Jiří Šponer,[2] Martin Ferus[1*] and Judit E. Šponer[2*]

[1]J. Heyrovský Institute of Physical Chemistry, Czech Academy of Sciences, Dolejškova 3, CZ 18223, Prague 8, Czech Republic

[2]Institute of Biophysics of the Czech Academy of Sciences, Královopolská 135, 61265 Brno, Czech Republic

[3]Department of Chemistry and Biochemistry, Mendel University in Brno, Zemědělská 1, CZ 613 00 Brno, Czech Republic

[4]Central European Institute of Technology, Brno University of Technology, Purkynova 123, CZ-612 00 Brno, Czech Republic

[5]Institute for Chemical-Physical Processes, Italian National Research Council (IPCF-CNR), Viale Ferdinando Stagno d’Alcontres 37, 98158 Messina, Italy

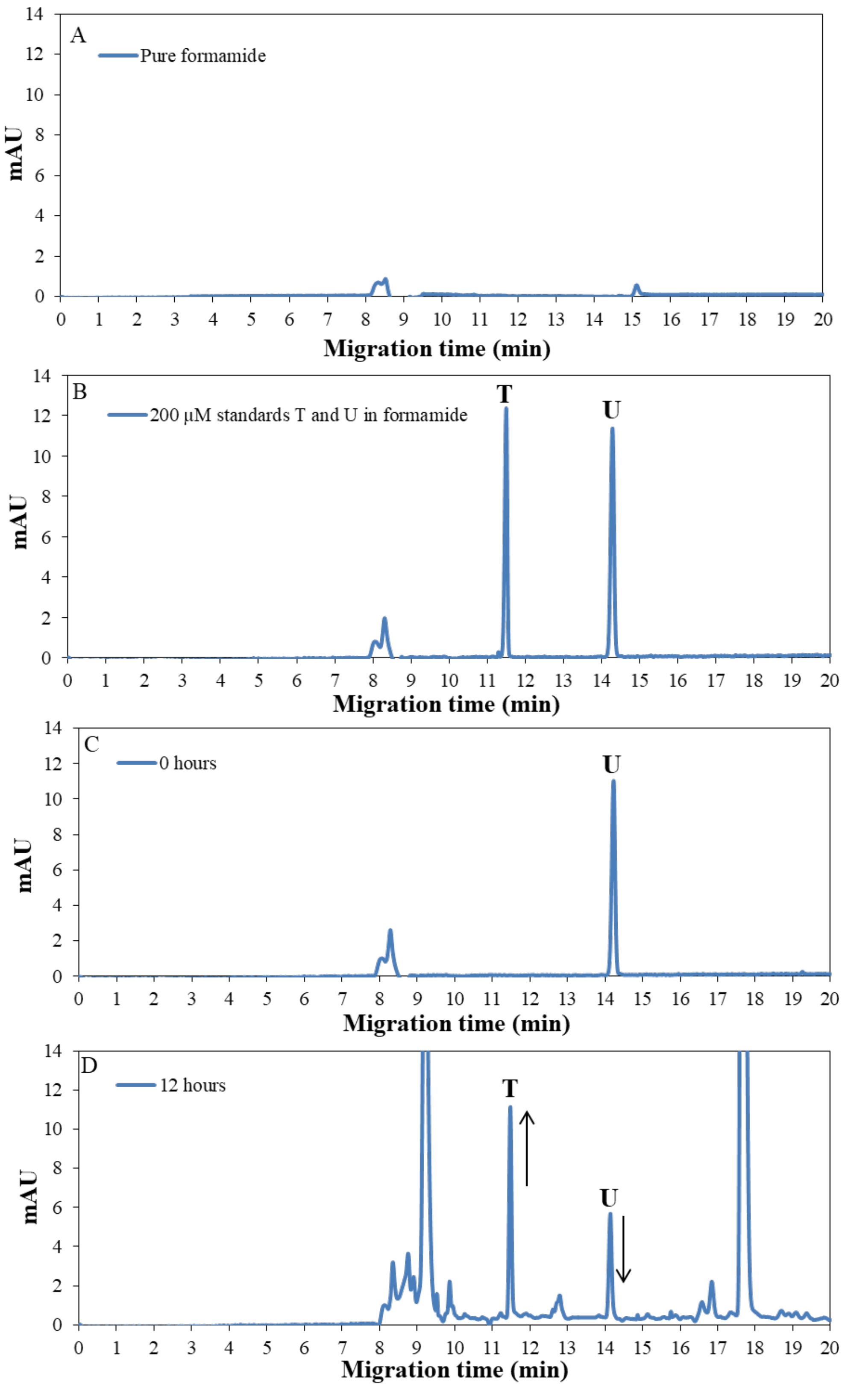

Figure S1: Separation of nucleobases thymine (T) and uracil (U) by CE. BGE: 40 mM sodium borate with 60 mM SDS (pH 9.8). Hydrodynamic injection: 50 mbar for 5 s, separation voltage: 15 kV, absorption signal at λ = 260 nm. Electropherograms of **(A)** pure formamide, **(B)** 200 µM standards T and U in formamide, **(C)** formamide sample with 2000 µM U before thermolysis (0 hours) and **(D)** after 12 hours of thermolysis.

Optimized geometries and computed electronic energies for all structures from Figure 3, main text.

structure 1 (uracil), B3LYP/6-31G* electronic energy (Hartree): -414.839207

N	-0.178893	-0.155479	0.575524
C	0.332176	0.168722	1.820444
O	-0.020908	1.157628	2.452904
N	1.277382	-0.731560	2.277197
C	1.746753	-1.887800	1.631080
O	2.598254	-2.592368	2.173427
C	1.139881	-2.124329	0.335409
C	0.211147	-1.259664	-0.137809
H	1.675714	-0.526803	3.188454
H	-0.876242	0.475903	0.198169
H	1.443917	-2.994038	-0.231708
H	-0.277300	-1.382554	-1.097632

structure 2, B3LYP/6-31G* electronic energy (Hartree): -529.367702

N	-0.006464	-0.004708	0.477130
C	0.411531	0.237260	1.773125
O	0.143162	1.264556	2.385077
N	1.162037	-0.795399	2.305224
C	1.522827	-2.001670	1.687672
O	2.214082	-2.818750	2.295460
C	1.028758	-2.155738	0.322845
C	0.288910	-1.151514	-0.212340
H	1.494205	-0.655302	3.254804
H	-0.559585	0.723768	0.041040
C	1.325657	-3.424869	-0.428352
H	-0.108895	-1.194887	-1.220793
H	1.127913	-3.271266	-1.497510
O	0.596963	-4.552997	0.071361
H	2.380249	-3.689914	-0.311817
H	-0.346890	-4.361799	-0.060114

structure 3, B3LYP/6-31G* electronic energy (Hartree): -642.709946

N	0.077549	0.085780	0.516412
C	0.366874	0.176380	1.868875
O	0.083338	1.153254	2.549524
N	1.007000	-0.944297	2.366558
C	1.373931	-2.101744	1.668216
O	1.954790	-3.018041	2.247939
C	1.015116	-2.093021	0.254127
C	0.385787	-1.000947	-0.252479
H	1.244453	-0.914485	3.353846
H	-0.386867	0.885568	0.100861
C	1.356331	-3.282555	-0.585413
H	0.096231	-0.924882	-1.295127
H	1.245276	-3.057246	-1.649524

O	0.524387	-4.436590	-0.243473
H	2.374441	-3.624205	-0.395513
C	-0.592046	-4.625124	-0.958343
H	-0.736243	-3.900248	-1.775573
O	-1.357831	-5.530640	-0.715452

structure 4, B3LYP/6-31G* electronic energy (Hartree): -454.131313

N	-0.216571	-0.172046	0.653766
C	0.361166	0.150685	1.837621
O	0.000837	1.073526	2.565781
N	1.421067	-0.688777	2.227237
C	1.789645	-1.895064	1.656664
O	2.593981	-2.635014	2.214759
C	1.126920	-2.192883	0.353128
C	0.431379	-1.040802	-0.331238
H	1.820674	-0.477879	3.136313
H	-0.886708	0.506643	0.312177
C	1.206162	-3.425658	-0.157184
H	1.162919	-0.464114	-0.918044
H	0.724062	-3.684049	-1.095621
H	1.763713	-4.202567	0.356478
H	-0.330475	-1.408900	-1.021468

structure 5, B3LYP/6-31G* electronic energy (Hartree): -454.514428

N	0.000569	0.051851	0.555912
C	0.424569	0.272267	1.829859
O	0.175719	1.265148	2.489932
N	1.201616	-0.779127	2.373996
C	1.573321	-1.967069	1.804541
O	2.239583	-2.826695	2.355676
C	1.069214	-2.150236	0.392122
C	0.339671	-1.081211	-0.239124
H	1.517138	-0.617652	3.328776
H	-0.533482	0.795963	0.120815
C	1.363333	-3.393982	-0.288521
H	1.032208	-0.803011	-1.076635
H	-0.527747	-1.481542	-0.794497
H	1.071090	-3.396307	-1.338387
H	0.802524	-4.179083	0.257483
H	2.415381	-3.671264	-0.134027

structure 6 (TS), B3LYP/6-31G* electronic energy (Hartree): -454.513642

N	-0.100235	0.000058	0.581205
C	0.380262	0.259244	1.841891
O	0.100723	1.246762	2.492791
N	1.236302	-0.739559	2.343962
C	1.629909	-1.917146	1.756862
O	2.362158	-2.736597	2.280259

C	1.027779	-2.158710	0.389581
C	0.286622	-1.099155	-0.194516
H	1.602280	-0.556136	3.276348
H	-0.669938	0.721028	0.151345
C	1.301442	-3.426031	-0.288443
H	1.334193	-0.914124	-0.706308
H	-0.364947	-1.319248	-1.042794
H	0.979788	-3.421710	-1.330704
H	0.709175	-4.180919	0.259874
H	2.349194	-3.719709	-0.173433

structure 7, B3LYP/6-31G* electronic energy (Hartree): -454.566478

N	-0.222422	-0.131067	0.577656
C	0.227297	0.089585	1.939494
O	-0.311502	0.930026	2.613161
N	1.259086	-0.723594	2.317954
C	1.902039	-1.701385	1.554897
O	2.798642	-2.366536	2.026519
C	1.413398	-1.870803	0.122570
C	0.289532	-0.987170	-0.242480
H	1.591367	-0.597411	3.272617
H	-0.998980	0.469103	0.288743
C	1.080315	-3.352679	-0.205270
H	2.244240	-1.572444	-0.539369
H	-0.143799	-1.039903	-1.238825
H	0.831087	-3.450355	-1.264513
H	0.241025	-3.702444	0.400827
H	1.963383	-3.954874	0.013940

structure 8 (thymine), B3LYP/6-31G* electronic energy (Hartree): -454.158855

N	-0.119468	-0.111207	0.570646
C	0.446285	0.233606	1.780445
O	0.188852	1.275167	2.376225
N	1.334356	-0.715670	2.253876
C	1.693075	-1.928895	1.651320
O	2.505625	-2.673022	2.203436
C	1.039425	-2.199665	0.375552
C	0.166487	-1.277563	-0.100262
H	1.775733	-0.503473	3.143432
H	-0.778690	0.550411	0.177928
C	1.365537	-3.479070	-0.341007
H	2.436466	-3.541059	-0.568944
H	0.807877	-3.552651	-1.278840
H	1.119828	-4.350726	0.277483
H	-0.360438	-1.402873	-1.039763

formaldehyde, B3LYP/6-31G* electronic energy (Hartree): -114.506883

C	-0.795053	-4.583845	-1.319591

O -1.063657 -5.056514 -2.404369
H -1.182637 -3.599620 -0.994792
H -0.147153 -5.109980 -0.593077

formic acid, B3LYP/6-31G* electronic energy (Hartree): -189.766497
H 0.819434 -5.606883 -1.066310
O 0.322520 -4.924999 -0.568063
C -0.765703 -4.592124 -1.272899
O -1.075872 -5.061045 -2.346376
H -1.340179 -3.827509 -0.733563

water, B3LYP/6-31G* electronic energy (Hartree): -76.420739
O 0.000000 0.000000 -0.018996
H -0.535846 0.535846 -0.625502
H 0.535846 -0.535846 -0.625502

carbon dioxide, B3LYP/6-31G* electronic energy (Hartree): -188.584272
C 0.177800 -0.120305 -0.079257
O 0.378076 0.314578 0.987080
O -0.022476 -0.555187 -1.145591

6
formamide, B3LYP/6-31G* electronic energy (Hartree): -169.904509
N 0.105906 -4.435505 -0.369358
C -0.806776 -4.368756 -1.356825
H -1.122593 -3.332181 -1.569587
O -1.258714 -5.333915 -1.972785
H 0.478971 -5.330612 -0.076129
H 0.435057 -3.600472 0.096024

7
protonated formamide, B3LYP/6-31G* electronic energy (Hartree): -170.324450
N 0.350935 -4.429801 -0.545384
C -0.865565 -4.355145 -1.435784
H -1.016764 -3.343702 -1.829565
O -1.520585 -5.327195 -1.629261
H 0.475197 -5.384964 -0.186649
H 0.263218 -3.784459 0.254223
H 1.201933 -4.155704 -1.059390

4
H3O+, B3LYP/6-31G* electronic energy (Hartree): -76.834834
O -0.159933 -0.085066 -0.087310
H -0.465208 0.710549 -0.585344
H 0.706749 -0.365260 -0.467558
H -0.011757 0.179187 0.851921
